# Infantile Botulism in the Absence of Dietary Exposure to Honey: A Case Likely Related to Environmental Spore Exposure

**DOI:** 10.7759/cureus.105728

**Published:** 2026-03-23

**Authors:** Kiley A Fincher, Eleanor Koonce-Oubre, Amy V Prudhomme

**Affiliations:** 1 Pediatrics, Louisiana State University Health Sciences Center, New Orleans, USA

**Keywords:** clostridium botulinum spores, honey-free botulism, infant botulism, infant environmental exposure, infant hypotonia, infant respiratory failure, urban infant botulism

## Abstract

Infantile botulism is a rare but potentially life-threatening neuroparalytic condition caused by botulinum toxin produced by *Clostridium botulinum *colonizing the infant's gastrointestinal tract. Early manifestations are often subtle and nonspecific, frequently leading to delayed or missed diagnosis. We report a case of a four-week-old infant who developed progressive hypotonia and respiratory compromise secondary to infantile botulism. The diagnosis was confirmed by detection of botulinum toxin in the stool, and the patient was treated promptly with botulism immune globulin. This case highlights the importance of considering neuromuscular etiologies such as botulism in infants presenting with atypical or unexplained respiratory decline. Increased awareness of its distinguishing features and early recognition are critical to prevent progression to respiratory failure.

## Introduction

*Clostridium* *botulinum* is a spore-forming, obligate anaerobic, Gram-positive bacillus that produces botulinum toxin [1]. Botulinum neurotoxins (BoNTs) are among the most potent toxins known and exert their effects by targeting motor neurons and blocking cholinergic neuromuscular transmission in multiple tissues, most critically the diaphragm, leading to respiratory failure [2]. BoNTs act by cleaving soluble N-ethylmaleimide-sensitive factor attachment receptor (SNARE) proteins, thereby inhibiting synaptic vesicle fusion with the presynaptic membrane and preventing acetylcholine release at the neuromuscular junction.

Botulism typically presents with cranial nerve palsies, followed by a descending, symmetrical pattern of weakness involving the trunk and limbs, as consistently described in clinical reviews [1]. In severe cases, this can result in generalized flaccid paralysis and significant respiratory compromise, requiring mechanical ventilation. Delayed diagnosis and treatment may be fatal. Prompt administration of botulinum antitoxin, along with supportive care, is essential for a favorable outcome [3].

Although uncommon, botulism occurs in approximately 100-150 individuals annually in the United States, most frequently affecting children under one year of age. *C. botulinum* spores are widely present in the environment, particularly in soil, river and lake sediments, and household dust. Their ability to withstand heat, drying, and chemical destruction allows them to persist in diverse environments, creating opportunities for infant exposure [2,4].

The differential diagnosis for infants presenting with hypotonia and respiratory decline is broad and includes spinal muscular atrophy, cerebral palsy, Guillain-Barré syndrome variants, metabolic disorders, sepsis, meningitis, mitochondrial disorders, and toxic exposures such as organophosphate and heavy metal poisoning [4-6]. Recognition of the progressive nature and distinguishing clinical features of botulism is crucial for timely diagnosis and appropriate management [2,6].

## Case presentation

A four-week-old full-term female (born at 39 weeks and six days) presented to an outside hospital (OSH) emergency department on February 28th with one day of feeding difficulties, nasal congestion, and increased somnolence. Her mother reported poor latch during breastfeeding, choking with bottle feeds, and decreased urine output. There were no reported fevers or known sick contacts.

On arrival at the OSH, the patient was hypoxic with grunting and bedside pulse oximetry reporting oxygen saturation levels in the 70s. Deep suctioning removed a large mucus plug with transient improvement, but she continued to have persistent desaturations to 70-80%, particularly with feeds, as well as postprandial emesis. She was placed on a low-flow nasal cannula at 1 L/min and transferred to our tertiary care center later that morning for further evaluation.

On initial exam at our hospital, the patient had a weak suck but otherwise exhibited age-appropriate muscle tone. The cardiopulmonary exam was unremarkable. Pupils were equal, round, and reactive to light. There were no focal neurological deficits, and the Moro reflex was symmetric.

**Figure 1:**
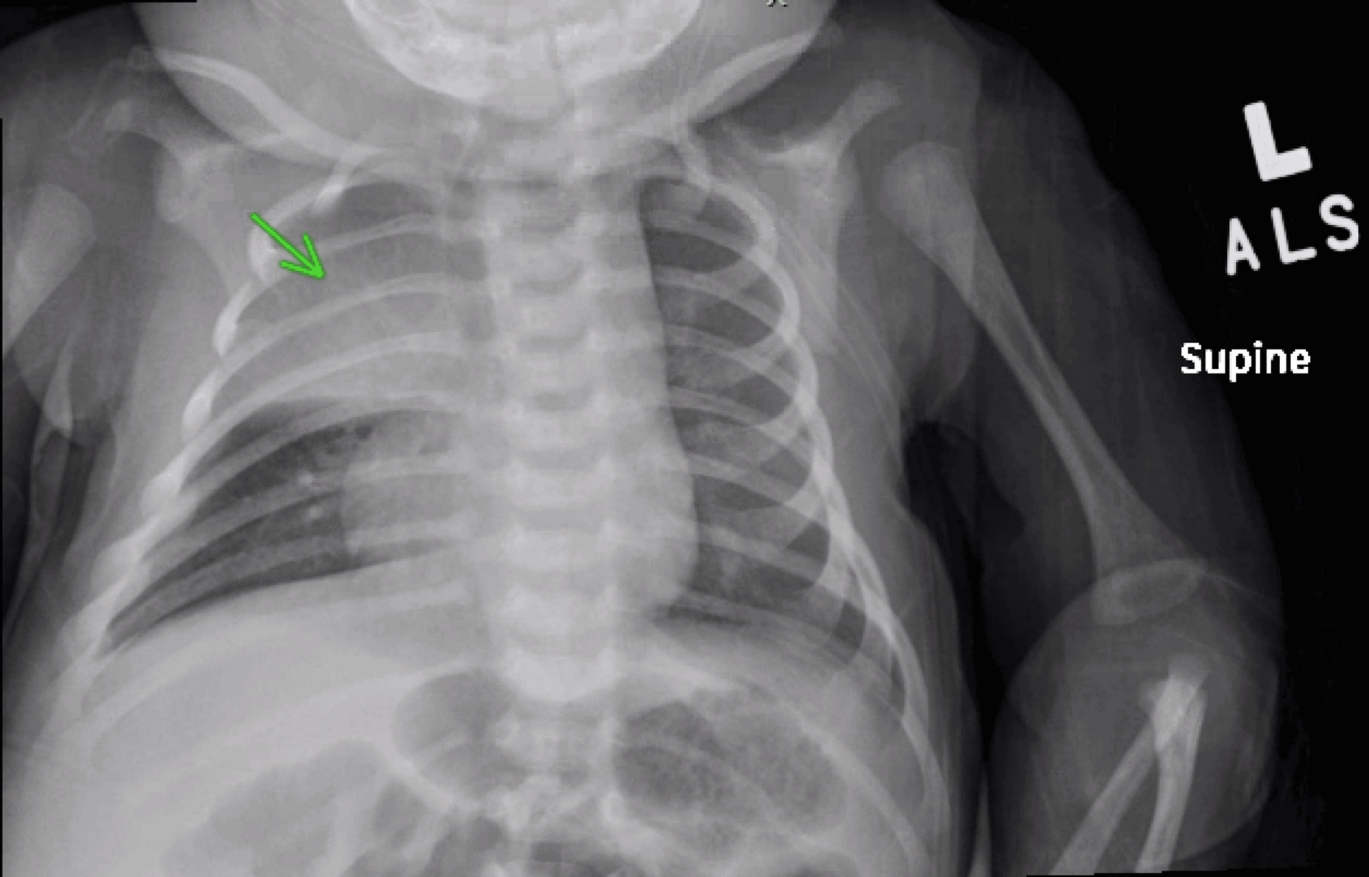
Chest X-ray on admission initially concerning for right upper lobe consolidation, later determined to represent a thymic shadow (arrow).

Laboratory and imaging studies were obtained. A respiratory viral panel was negative. Chest radiography initially showed right upper lobe (RUL) consolidation, but later revealed a prominent thymic shadow (Figure 1). A nasogastric tube was placed to administer continuous Pedialyte® feeds (Abbott Laboratories, Abbott Park, IL, USA). A full infectious workup was performed, including blood culture, a complete blood count, a comprehensive metabolic panel, procalcitonin, C-reactive protein, urinalysis, and urine culture, and empiric intravenous ceftriaxone was started. The patient was admitted for ongoing oxygen requirement and concern for pneumonia.

Later that same morning, the care team was urgently called to the bedside due to concern for an altered mental status. The infant was noted to have a weak cry, minimal response to painful stimuli, generalized hypotonia, bradypnea with intermittent apneic episodes, prolonged capillary refill, and mottled skin. Laboratory studies at that time demonstrated a serum bicarbonate level of 17 mmol/L (18-30) and a chloride level of 108 mmol/L (98-107); all other parameters were within normal limits. A capillary blood gas showed a pH of 7.31 (7.35-7.45), a pCO_2_ of 60 mmHg (35-45), and a bicarbonate level of 30.2 mmol/L (22-26). Repeat chest radiograph showed no interval change. Due to clinical deterioration and metabolic acidosis, she was escalated to a high-flow nasal cannula at 10 L/min and promptly transferred to the pediatric intensive care unit (PICU).

In the PICU, she remained on empiric ceftriaxone and was started on empiric vancomycin due to concern for an infectious etiology. A head ultrasound demonstrated a resolving grade 1 germinal matrix hemorrhage, and a subsequent CT scan showed no acute intracranial abnormalities. Brain magnetic resonance imaging (MRI) and plasma amino acid profiling were performed and were noncontributory. Lumbar puncture findings were unremarkable, and a urine drug screen was negative. She was initially supported with noninvasive positive pressure ventilation; however, she continued to experience recurrent desaturation episodes and pooling of oral secretions, leading to upper airway obstruction. She was subsequently intubated on hospital day 2, during which an absent gag reflex was noted. An endotracheal tube (ETT) culture collected at that time returned positive for *Staphylococcus aureus* and *Enterobacter cloacae*. Ceftriaxone was transitioned to cefepime, and she was continued on vancomycin for a seven-day course of antibiotics. These findings, however, did not account for her depressed mental status, hypotonia, or bulbar dysfunction.

Given the persistence of hypotonia and bulbar dysfunction, including dysphagia, absent gag reflex, and weak cry, clinical suspicion for infantile botulism was raised. Stool and serum samples were sent for confirmatory testing, and botulism immune globulin intravenous (BIG-IV, BabyBIG®; California Department of Public Health, Richmond, CA, USA) was administered on hospital day 3. The case was reported to the Centers for Disease Control and Prevention (CDC).

Further history revealed that the infant had been exclusively fed breast milk and formula, with no exposure to honey. The family resided in a nonagricultural area with no known nearby construction; however, the mother reported taking the infant for a walk in a public park several days prior to symptom onset.

Over the ensuing days after receiving BIG-IV, the patient demonstrated progressive improvement in her neurological exam (see Table 1). She was successfully extubated on hospital day 11 and transferred to the general pediatric floor. Serum testing was positive on hospital day 15. While on the general floor, the patient tolerated full oral feeds without nasogastric support, achieved appropriate weight gain, and was discharged home in stable condition. A stool specimen, analyzed for botulinum toxin serotypes A, B, E, and F via BoNT endopeptidase-mass spectrometry, resulted positive for BoNT type B on April 28th, approximately five weeks after discharge.

**Table 1:** Detailed PICU course. PICU: pediatric intensive care unit; NC: nasal cannula; HFNC: high-flow nasal cannula; NIPPV: noninvasive positive pressure ventilation; PEEP: positive end-expiratory pressure; FiO_2_: fraction of inspired oxygen; PIP: peak inspiratory pressure; PC: pressure control; BID: twice daily; TID: three times daily; ETT: endotracheal tube; CPAP: continuous positive airway pressure; RA: room air; BIG-IV: botulism immune globulin intravenous

Hospital Day	Neuro Exam/Major Events	Respiratory Settings
1	Weak suck, weak cry, generalized hypotonia, minimal response to stimuli	NC 1 L/min → HFNC 10 L/min → NIPPV PEEP of 5 cmH_2_O, FiO_2_ of 30%, and pressure support of 10 cmH_2_O
2	Persistent hypotonia, pooling of oral secretions	NIPPV → Intubation and mechanical ventilation (PC): PIP 16/PEEP 6
3	Exam unchanged from day 2; BIG-IV administered 19:30	Mechanical ventilation (PC): PIP 16/PEEP 6
4	Sporadic movement of hands, feet, and head; still not opening eyes; movements limited and less coordinated	Mechanical ventilation (PC): PIP 18/PEEP 5; vent sprints two hours BID, tachypneic and tachycardic at the end of the sprint
5	Increased spontaneous movement: hands, feet, knees, elbows, head, mouth	Mechanical ventilation (PC): PIP 15/PEEP 5; vent sprints two hours TID, tachypneic and tachycardic at the end of each sprint
6	Spontaneous movement; intermittently fighting ETT; eyes briefly opened	Mechanical ventilation (PC): PIP 15/PEEP 5; vent sprints three hours BID, tachypnea/tachycardia not observed
7-8	Significantly more responsive, able to keep eyes open, biting the ETT	Day 7 - vent sprints three hours TID; Day 8 - vent sprints four hours TID; Day 9 - daily CPAP, nighttime rate support
9-10	Improved alertness and spontaneous movement, preparing for extubation	CPAP mode x 24 hours
11	Extubated, tolerated well	0.5L NC → RA

## Discussion

According to recent CDC data, infantile botulism is the most frequently reported form of botulism in the United States. In 2019, a total of 215 botulism cases were reported to the CDC. Of these, 152 cases (approximately 70%) were infantile botulism. Other forms, such as wound, foodborne, and adult intestinal colonization, accounted for a much smaller proportion [3]. More recent data show a similar pattern. In 2020, the United States reported 226 botulism cases, including 159 infants (70%). In 2021, surveillance documented 273 total cases, with 181 (66%) being infant botulism [7,8].

While these data highlight infantile botulism as the predominant form of the disease, it remains a relatively rare condition. Notably, no deaths were reported among the 2019 cases, pointing to a low mortality rate with timely diagnosis and appropriate treatment [4]. However, delayed recognition can lead to significant morbidity. For example, a 30-year retrospective study conducted in the ICU at Children's Hospital Los Angeles demonstrated that infants who did not receive BIG-IV had a median hospital stay of approximately 35 days, including 24 days in the ICU and 17 days requiring mechanical ventilation. In contrast, infants who received BIG-IV had median reductions of approximately 50% in hospital and ICU stays, and nearly 60% in mechanical ventilation duration, compared with untreated patients during the same period [9]. BIG-IV, approved by the U.S. Food and Drug Administration (FDA), contains human plasma-derived antibodies that neutralize circulating botulinum toxin. More than 2,180 infants in the United States have received this therapy, collectively reducing hospital stays by over 128 years and saving an estimated $174 million in hospital-related costs [10]. Consistent with these findings, the 2024 CDC guidelines recommend administering BIG-IV as soon as infant botulism is suspected, ideally within 10 days of symptom onset, even before laboratory confirmation [9].

Despite the clear benefits of early treatment, diagnosis remains challenging. Initial symptoms - constipation, poor feeding, hypotonia, and weakness - are nonspecific and may mimic common pediatric issues. This often leads to treatment delays and increases the risk of complications, such as prolonged ventilation and longer ICU stays. To address this, healthcare providers need greater awareness to support early recognition, accurate diagnosis, and prompt therapy. Preventive education is also key, especially in community settings. In addition to advising caregivers not to feed honey to infants younger than 12 months, education should also address potential exposure to *C. botulinum* spores in dust, soil, and certain home-prepared foods or syrups. Stressing hand hygiene, safe environmental practices, and early symptom recognition enables timely care. Working with pediatric providers, community groups, and digital campaigns can improve message reach and impact in at-risk communities.

With timely recognition and appropriate management, infants with botulism can achieve full recovery, as demonstrated by the present case. At follow-up approximately six months after discharge, the patient exhibited normal muscle tone, adequate feeding, and age-appropriate neurodevelopmental milestones, with no evidence of persistent neuromuscular weakness or developmental delay. Although long-term outcomes in infant botulism are generally favorable, potential sequelae reported in the literature include transient hypotonia, delayed motor milestone attainment, feeding difficulties, and complications related to prolonged hospitalization or mechanical ventilation [11,12]. Nonetheless, most infants who receive timely treatment recover fully without permanent neurologic impairment.

## Conclusions

Infantile botulism can initially present with nonspecific symptoms such as feeding difficulties, hypoxia, and respiratory distress, which often lead to misdiagnosis as pneumonia or another infectious process. This case highlights the importance of careful history-taking and targeted questioning to support accurate diagnosis, particularly when initial presentations are atypical. Early recognition and prompt administration of BIG-IV are essential to improving outcomes, as timely treatment has been shown to significantly reduce hospital and ICU length of stay and the need for mechanical ventilation. Notably, this case is especially compelling given the absence of any known exposure, emphasizing the diagnostic challenge and the need to maintain clinical suspicion even when classic risk factors are not apparent. Continued vigilance and awareness are necessary, as infantile botulism can occur without identifiable environmental or dietary exposures, and timely intervention remains the cornerstone of a favorable outcome.
